# The Effect of Arginase on Canine T-Lymphocyte Functions and its Modulation by All-Trans Retinoid Acid (ATRA) in Canine Monocyte-Derived Macrophages

**DOI:** 10.3390/vetsci9070374

**Published:** 2022-07-21

**Authors:** Aimee M. Soileau, Cally N. Quick, Cambri E. Moeller, Jessica C. Schaumburg, Sita S. Withers

**Affiliations:** Department of Veterinary Clinical Sciences, School of Veterinary Medicine, Louisiana State University, 1909 Skip Bertman Dr., Baton Rouge, LA 70803, USA; aimee.m.soileau@gmail.com (A.M.S.); callyquick@gmail.com (C.N.Q.); cmoeller1@lsu.edu (C.E.M.); jschau3@lsu.edu (J.C.S.)

**Keywords:** canine, ATRA, myeloid cells, tumor associated macrophages, cancer, oncoimmunology, T-lymphocytes

## Abstract

**Simple Summary:**

Vast improvements in our understanding of the host’s immune response to tumor cells has dramatically improved cancer treatment in the last 20 years. Naturally occurring cancers in domesticated dogs closely model the immune response to cancers in humans. Therefore, understanding similarities and differences between the immune systems of domesticated dogs and people is critical to translating results relating to immune-targeting cancer therapies between species. To this end, this study evaluated the ability of all-trans retinoic acid, a vitamin A derivative, to decrease the production of pro-cancer substances from a particular immune cell (macrophages). These macrophages were developed in vitro from the blood of dogs with naturally occurring cancers. Our data revealed the ability of all-trans retinoic acid (ATRA) to decrease macrophage production of substances that have been previously reported to increase tumor cell invasion and spread as well as inhibit an effective anti-tumor immune response. We also confirmed the inhibitory activity of one of these substances on critical anti-cancer immune cells, T-lymphocytes. While additional studies are needed to show a direct link between ATRA treatment, macrophage inhibition, and subsequent T-lymphocyte stimulation, these preliminary findings suggest a potential role for ATRA in modulating cancer immunity in dogs.

**Abstract:**

Immunosuppressive myeloid cells in the tumor microenvironment play a major role in suppressing tumor immunity via the production of arginase, IL-10, and others. The objectives of this study were to determine the ability of all-trans retinoic acid (ATRA) to decrease the expression of arginase and other soluble mediators by canine monocyte-derived macrophages (MDMs) and to determine the inhibitory activity of arginase on canine T-lymphocytes. The immunomodulatory ability of ATRA (2 µM) on canine MDMs was evaluated via reverse transcription quantitative PCR (RT-qPCR), flow cytometry, arginase activity assay, and enzyme-linked immunoassay (ELISA). Arginase effects on T-lymphocyte phenotype and proliferation were then evaluated by flow cytometry. ATRA consistently decreased MDM expression of IL6, TGFB1, NOS2, ARG1, and CIITA transcripts, by approximately 2–4-fold, although this did not reach statistical significance for ARG1 or CIITA. Furthermore, arginase activity was decreased in ATRA-treated MDMs while the MDM phenotype remained unchanged. Arginase decreased the expression of granzyme B on CD8+ T-lymphocytes and inhibited CD4+ and CD8+ T-lymphocyte proliferation. These findings suggested that ATRA could inhibit canine MDM production of soluble inflammatory/immunosuppressive mediators. These data also revealed that arginase decreased canine T-lymphocyte proliferation and granzyme B expression. Further studies are needed to determine whether ATRA could reverse the immunosuppressive effects of myeloid cells on canine T-lymphocytes in vivo.

## 1. Introduction

L-arginine (L-arg) is an essential amino acid in dogs and has been shown to play a significant role in supporting T-lymphocyte and natural killer (NK) cell function in humans and mice [1,2,3,4,5]. Arginase metabolizes L-arg to L-ornithine and urea and is expressed by immunosuppressive myeloid cells such as M2-type macrophages and myeloid-derived suppressor cells (MDSCs). Tumor-associated macrophages (TAMs) and MDSCs within the tumor microenvironment (TME) exhibit increased arginase expression in humans and mouse models, and the inhibitory effects of arginase on T-cell lymphocyte function have been documented in these species [3,4,6]. The expression of arginase mRNA has been documented in MDSCs in cancer-bearing dogs; however, the direct effect of arginase on canine T-lymphocytes has not been evaluated [7].

In addition to arginase, various other soluble mediators expressed by immunosuppressive myeloid cells have been shown in human studies to contribute to tumor immunoevasion, such as IL-10, TGF-β1, IL-6, and inducible nitric oxide synthase (iNOS) [8,9,10]. IL-10 and TGF-β1 inhibit T-lymphocyte function in the TME and have been considered key immunosuppressive factors released by TAMs, MDSCs, and tumor cells [9,11,12,13]. High levels of IL-6 have been associated with a poor prognosis in various human cancers, and within the TME, IL-6 promoted cancer cell proliferation, angiogenesis, and immunosuppression [12,14]. iNOS caused the formation of harmful reactive oxygen species that could promote carcinogenesis through DNA damage and inhibit DNA repair and apoptosis [15]. iNOS also promoted cancer progression by increasing cancer cell proliferation, migration, and tumor angiogenesis [15]. Major histocompatibility complex II (MHCII) molecules are responsible for presenting antigens to CD4+ T-lymphocytes and are therefore critical in the antigen-specific immune response. Cell surface expression of MHCII could be significantly low-to-absent in immunosuppressive myeloid cells, compromising their ability to present antigen [16,17]. 

All-trans retinoic acid (ATRA), a vitamin-A derivative used to treat leukemia and dermatological conditions in humans, has been shown to differentiate TAMs and MDSCs into mature dendritic cells, macrophages, and granulocytes in humans and mice [18,19,20,21]. Furthermore, the treatment of human monocytic MDSCs with ATRA revealed a decrease in the expression of immunosuppressive soluble mediators, including IL-10 and TGF-β1, and an increase in MHCII expression [21]. In a syngeneic mouse model of osteosarcoma, ATRA treatment decreased the proportion of cancer cells with “stemness” properties by inhibiting the M2 polarization of TAMs [20]. The effect of ATRA on arginase expression by myeloid cells remains unclear, however. Mouse bone marrow cells exposed to IL-4 increased their expression of arginase upon exposure to ATRA [22,23]. Therefore, the cumulative effect of ATRA may depend on the subset of myeloid cells and other microenvironmental factors. With several clinical trials underway evaluating ATRA as an immunomodulator in human cancers (www.clinicaltrials.gov (accessed on 11 November 2021)), an understanding of how ATRA modulates canine myeloid cells is necessary for future comparative studies.

The primary objective of this study was to evaluate the ability of ATRA to modulate canine monocyte-derived macrophages (MDMs) by altering the expression of arginase and other soluble factors. Secondarily, we defined the inhibitory effects of arginase on canine T-lymphocytes. We hypothesized that ATRA would decrease the expression of various immunosuppressive soluble mediators (e.g., IL-10, TGF-β1, and arginase) by differentiating canine MDMs and that arginase would decrease canine T-lymphocyte functionality. These data would establish a preclinical foundation for determining the immunomodulatory activity of ATRA in clinical canine cancer.

## 2. Materials and Methods

### 2.1. Study Population and Sample Collection

Peripheral blood samples were collected from client-owned dogs presenting with cancer at Louisiana State University, School of Veterinary Medicine (LSU SVM). A total of 15 mL was collected from the jugular vein of dogs weighing > 15 kg after informed consent was granted by their owners. All sample collections were performed in accordance with an approved Institutional Animal Care and Use Committee (IACUC) protocol (#19-068). Included dogs did not exhibit clinical signs, including lethargy, inappetence, vomiting, or diarrhea, and had not had blood collected for research purposes within the previous four weeks. All blood was processed on the day of collection. Signalment and tumor burden of each patient are summarized in the Supplementary Table S1. 

### 2.2. Reagents

Peripheral blood samples were diluted at a ratio of 1:1 in Dulbecco’s phosphate buffered saline (DPBS, Gibco, Carlsbad, CA, USA) and layered over Histopaque 1077 (Sigma-Aldrich, Saint Louis, MO, USA). A 1× red blood cell (RBC) lysis buffer (420301, Biolegend, San Diego, CA, USA) was used for RBC elimination. Peripheral blood mononuclear cells (PBMCs) were cultured *in vitro* in complete media, which included 10% fetal bovine serum (FBS, heat-inactivated, Omega Scientific, Tarzana, CA, USA), 5% canine serum (heat-inactivated, SCA32-0500, Equitech-Bio, Kerrville, TX, USA), and 1% penicillin/streptomycin (P/S; 100 U/mL penicillin and 100 μg/mL streptomycin, Gibco, Carlsbad, CA, USA) in Roswell Park Memorial Institute media (RPMI 1640; Gibco, Carlsbad, CA, USA). For evaluation of the effects of arginase on T-lymphocyte phenotype and proliferation, cells were treated with arginase (derived from bovine liver, A3233, Sigma-Aldrich, St. Louis, MO, USA) at increasing concentrations (25 nM, 50 nM, and 100 nM), and for the proliferation experiment, Concanavalin A (ConA, C5275, Sigma-Aldrich, St. Louis, MO, USA) was used to stimulate cells at a concentration of 5 μg/mL. 

ATRA (0695/50, R&D Systems, Bristol, UK) was dissolved in dimethyl sulfoxide (DMSO, D128-500, Fisher Scientific, Fair Lawn, NJ, USA) and added to cell cultures at increasing concentrations (1 μM, 2 μM, and 10 μM) to determine its cytotoxic effects, and a concentration of 2 μM was used for remaining ATRA-related experiments. For differentiation of MDMs, cells were incubated with human recombinant macrophage-colony-stimulating factor (rhM-CSF, *E. coli* derived, 216-MC, R&D Systems, Minneapolis, MN, USA) at a concentration of 25 ng/mL. A cold solution of 5 mM ethylenediaminetetraacetic acid (EDTA, AM9260G, Invitrogen, Burlington, ON, Canada) in DPBS was used to dislodge adherent MDMs for flow cytometry experiments, while 0.25% Trypsin-EDTA (trypsin, 25200-056, Gibco, Grand Island, NY, USA) was used to detach MDMs for all remaining experiments. 

Fixable viability dye (LIVE/DEAD Fixable Near-IR Dead Cell Stain Kit, Invitrogen, Eugene, OR, USA) was used to identify live cells for each flow cytometry experiment at a dilution of 1:1000 in DPBS. Surface antibody staining of PBMCs and MDMs was performed on cells suspended in a staining buffer, consisting of 3% FBS and 1 mM of EDTA in DPBS. Intracellular staining of PBMCs was performed using fixation/permeabilization concentrate and diluent (00-5123-43 and 00-5223-56, eBioscience, Carlsbad, CA, USA) and permeabilization buffer (00-8333-56, eBioscience, Carlsbad, CA, USA). The details of antibodies used in these studies and the references justifying non-commercial antibodies and antibodies directed against non-canine antigens are listed in Supplementary Table S2. 

### 2.3. PBMC Isolation 

Whole blood was diluted 1:1 in DPBS and layered over Histopaque 1077. The samples were centrifuged at 400× *g* rcf for 30 min at 4 °C with the deceleration set to low. PBMCs were then retrieved, transferred to a clean tube, and washed with DPBS. Cell preparations were centrifuged again at 400× *g* rcf for 15 min at 4 °C to pellet the cells. Red blood cells were then eliminated by incubating with 1× RBC lysis buffer for 3 min on ice and protected from light. PBMCs were washed again with DPBS, centrifuged at 400× *g* rcf for 10 min at 4 °C, and counted. 

### 2.4. In Vitro MDM Generation, Treatment and Collection 

After isolation, PBMCs were plated at a density of 1.0 × 10^6^ cells/well in a 96-well plate for cytotoxicity evaluation, and 5.0–8.0 × 10^6^ cells/well in a 6-well plate or 50–100 × 10^6^ cells in a 10 cm dish for the remaining MDM experiments. PBMCs were then incubated (37 °C, 5% CO_2_) in complete media overnight. The following day, media was gently removed and replaced with complete media containing rhM-CSF (25 ng/mL) to elicit M2 macrophage differentiation, as well as ATRA or DMSO [21,24,25]. After 2 days, cells were gently washed in DPBS and fresh media containing rhM-CSF and ATRA or DMSO was replaced. After a total of 5 days of differentiation in rhM-CSF, with or without ATRA, adherent cells were detached using EDTA or trypsin, in preparation for flow cytometry and remaining MDM experiments, respectively.

### 2.5. Cell Viability Assay

After 5 days of differentiation in rhM-CSF with increasing concentrations of ATRA or vehicle control media (DMSO only), cell viability of MDMs was assessed using an MTS assay, per the manufacturer’s protocol (CellTiter 96, One Solution Cell Proliferation Assay, Promega, Madison, WI, USA). The absorbance at 490 nm was recorded in each well using an Epoch 96-well plate spectrophotometer (BioTek, Winooski, VT, USA). Results were expressed as % viability relative to DMSO control.

### 2.6. Reverse Transcription-Quantitative PCR (RT-qPCR)

After MDM collection, cells were centrifuged at 400× *g* rcf for 5 min at 4 °C and resuspended in DPBS. RNA extraction was performed using the RNeasy Mini Kit (74104, Qiagen, Hilden, Germany), according to the manufacturer’s protocol. RNA was quantified using the Nanodrop One Spectrophotometer (Thermo Fisher Scientific, Waltham, MA, USA). Total RNA was stored at −80 °C. 

RNA was converted into cDNA using the qScript cDNA Synthesis Kit (95047-100, Quanta BioSciences, Gaithersburg, MD, USA), according to the manufacturer’s protocol. Total RNA (1 μg), qScript reagent (4 μL), and RT (1 μL) were aliquoted into 0.2 mL sterile PCR reaction tubes. The total reaction volume was up to 20 μL with nuclease-free water. The cDNA reaction tubes were incubated for 5 min at 25 °C, 30 min at 42 °C, 5 min at 85 °C, and then held at 4 °C in the thermal cycler. RT-qPCR reactions were prepared according to the manufacturer’s protocol using PerfeCTa SYBR Green FastMix ROX (95073-012, Quanta BioSciences, Gaithersburg, MD, USA). Primer pairs targeting ARG1 (arginase), NOS2 (iNOS), IL6 (IL-6), IL10 (IL-10), TGFB1 (TGF-β1), and class II major histocompatibility complex transactivator (CIITA) are listed in Supplementary Table S3. CIITA was included as a marker of MHCII expression [21]. The reactions were conducted in a 384-well plate with a total volume of 10 μL in each well, consisting of 2 uL of cDNA, 0.4 μL of forward and reverse primers, 5 μL of PerfeCTa SYBR Green FastMix, and 2.2 μL of nuclease-free water.

The reactions were determined by a 7900 HT Fast Real-Time PCR System (Applied Biosystems, Waltham, MA, USA), using recommended cycle conditions for PerfeCTa SYBR Green FastMix ROX kit: hold at 95 °C for 60 s, then from 40 cycles of 95 °C for 15 s to 60 °C for 60 s, and finishing with a dissociation curve. Reactions were performed in duplicate. RPS5 and C12orf43 were selected as reference genes [26,27]; however, RPS5 was routinely downregulated in ATRA-treated cells so C12orf43 was used for ΔCT calculations. A no-template control reaction was performed on each plate for all primer pairs to ensure no DNA contamination of reagents. Amplification of a single product was confirmed by evaluation of each dissociation curve, and the relative expression of ATRA-treated MDMs was compared to control MDMs via the ΔΔCT method. The fold-change was quantified using the formula 2^−ΔΔCT^. Fold-change was Log_2_-transformed for statistical analyses. 

### 2.7. Arginase Activity Assay

Detached MDMs were centrifuged at 400× *g* rcf for 5 min at 4 °C. Cells were resuspended in DPBS and counted. A total of 0.5–1 × 10^6^ MDMs were input into the arginase activity assay. Exact cell numbers varied between dogs, but cell numbers for ATRA-treated and control MDMs were equal for each dog. Arginase activity of cell lysates was then evaluated using an arginase activity assay kit, per manufacturer’s protocol (MAK112, Sigma-Aldrich, St. Louis, MO, USA). Absorbance (*A*) of diluted samples (1:8), non-diluted samples, and controls were read at 430 nM every 30 min for 4 h. Values were entered into the equation below to determine arginase activity:

(1) $$Activity=\frac{{\left(A_{430}\right)}_{sample}-{\left(A_{430}\right)}_{blank}}{{\left(A_{430}\right)}_{standard}-{\left(A_{430}\right)}_{water}}\times \frac{\left(1\;\mathrm{mM}\;\times 50\times 10^{3}\right)}{\left(V\times T\right)}$$

where 1 mM represents urea standard concentration, 50 represents reaction volume (µL), 10^3^ represents mM to µM conversion factor, *V* represents sample volume (µL) added to well, and *T* represents reaction time in minutes. Activity levels measured at 2 h were reported.

### 2.8. Enzyme-Linked Immunosorbent Assay (ELISA)

After MDM collection, cells were centrifuged at 400× *g* rcf for 5 min at 4 °C. Cells were then resuspended in DPBS, counted, and centrifuged at 400× *g* rcf for 5 min at 4 °C for a second time. Next, cells were plated at a density of 1.0 × 10^5^ cells/well in a 96-well plate in complete media containing rhM-CSF for 24 h. Supernatant was then centrifuged at 2000× *g* rcf for 5 min at 4 °C, aliquoted, and frozen at −80 °C. The TGF-β1 concentrations in supernatant from ATRA-treated and control MDMs were then measured using a multi-species TGF-β1 ELISA kit, per the manufacturer’s protocol (DB100C, Quantikine, R&D Systems, Minneapolis, MN, USA). Samples were diluted at a ratio of 1:10, and absorbance values from complete media were subtracted from MDM supernatants to account for the background. TGF-β1 concentration was then calculated using a standard curve. 

### 2.9. Cell Staining for Flow Cytometry

Firstly, cells were washed in DPBS and stained with viability dye for 30 min in a total volume of 250 μL. Samples were then washed again in DPBS. For staining of MDMs, samples were incubated with an Fc block diluted in staining buffer for 15 min. Cells were then immediately stained with a cocktail of surface antibodies diluted in staining buffer for 20 min, before being washed in staining buffer. For experiments requiring biotinylated anti-CD11b, subsequent incubation with streptavidin, conjugated to BV421, was required for 20 min, followed by another wash with staining buffer. In experiments with intracellular antibody staining, samples were then incubated in 250 μL of fixation/permeabilization solution for 20 min. Permeabilization buffer was then used to wash cells before incubation with a cocktail of intracellular antibodies diluted in permeabilization buffer. Finally, all samples were washed again in permeabilization buffer and then suspended in staining buffer. All samples were analyzed within 2 h of complete staining. All antibody staining steps were performed in a total volume of 50 μL per sample. All incubation steps were performed at 4 °C.

### 2.10. MDM Phenotyping

Following the collection of MDMs, cells were stained for CD5, CD11b, CD14, MHCII, and CD80 for flow cytometry analysis (Supplementary Table S2). Fluorescence-minus-one (FMO) controls were performed for CD11b and CD80 to assist in determining the threshold for positivity of these markers. Due to the difficulty in obtaining a high cell count, CD5+ T-lymphocytes were used as internal negative controls (INCs) for determining the expression of MHCII and CD14. Cells were gated on the myeloid cell cloud, single cells, and CD5-cells (Supplementary Figure S1A). CD5− MDMs, treated with or without ATRA, were evaluated for expression of median fluorescence intensity (MFI) of CD11b, CD14, MHCII, and CD80.

### 2.11. T-Lymphocyte Phenotyping

PBMCs were plated at a density of 1 × 10^6^ cells/well in a 96-well plate in complete media, with or without increasing concentrations of arginase, and incubated (37 °C, 5% CO_2_) for 2 days [28]. Cells were then stained for viability, CD3, CD4, CD8, and granzyme B (GZMB) for flow cytometry analysis (Supplementary Table S2). Cells were gated on single cells, viable cells, CD3+, CD4+CD8−, and CD4−CD8+ (Supplementary Figure S1B). FMO controls were used to gate GZMB+ cells. CD8+ T-lymphocytes were evaluated for expression of GZMB, and the %GZMB+ CD8+ T-cells was reported in arginase-treated cells relative to control. The MFI of CD3 was also compared between arginase-treated and control PBMCs.

### 2.12. Assessment of T-Lymphocyte Proliferation

PBMCs were plated at a density of 1 × 10^6^ cells/well in a 96-well plate in either complete media, complete media containing ConA, or complete media containing ConA and increasing concentrations of arginase, and incubated for 2 days. Next, cells were stained for viability, CD3, CD4, CD8, CD25, and Ki67 for flow cytometry analysis (Supplementary Table S2). Cells treated with only ConA and unstimulated cells served as biological positive and negative controls, respectively, in order to establish appropriate gating for the proliferation marker, Ki67. Cells were gated on single cells, viable cells, CD3+, CD4+CD8−, and CD4−CD8+ (Supplementary Figure S1C). CD4+ and CD8+ T-lymphocytes were evaluated for expression of Ki67 and the %Ki67+ T-cell subsets in arginase-treated PBMCs was compared to that of ConA-only treated PBMCs. The MFI of CD25 was also compared between arginase-treated and ConA-only treated PBMCs.

### 2.13. Flow Cytometry Data Acquisition and Analysis

Flow cytometry was performed on a Becton Dickinson LSRFortessa X-20 flow cytometer, utilizing violet (405 nM), blue (488 nM), yellow–green (561 nM), and red (640 nM) lasers. Compensation settings were conducted using BD CompBeads (BD Biosciences). Flow cytometry was analyzed using FlowJo software (Version 10; BD Biosciences, Ashland, OR, USA).

### 2.14. Statistics

Prism 9 for MacOS v9.1 (GraphPad Software) was used for statistical analyses. Two-tailed *t*-tests were used to determine significant differences between groups for the RT-qPCR, arginase activity assay, and ELISA results. For comparison of RTqPCR findings between groups, Log_2_(fold-change) was used to derive *p*-values. A one-way ANOVA with post hoc multiple comparisons test was used to determine significance for the T-lymphocyte phenotype and proliferation assays. Two biological replicates were performed for the MDM viability assay, and at least three biological replicates were performed for remaining experiments. The *p*-values < 0.05 were considered statistically significant and were stated as an equality for values > 0.001. The *p*-values and descriptive statistics for all comparisons made are listed in Supplemental Table S4.

## 3. Results

### 3.1. Effects of ATRA on Differentiating Canine MDM Viability

MDMs from two dogs were incubated with rhM-CSF and increasing concentrations of ATRA for five days to determine the potential effects of ATRA on cell viability. ATRA-treated MDMs from both dogs remained viable after 5 days of exposure to 2 µM (Supplementary Figure S2). Given these findings and those of a previous publication, we elected to proceed with the non-cytotoxic concentration of 2 µM [21].

### 3.2. mRNA and Protein Expression in ATRA-Treated Canine MDMs

**Figure 1:**
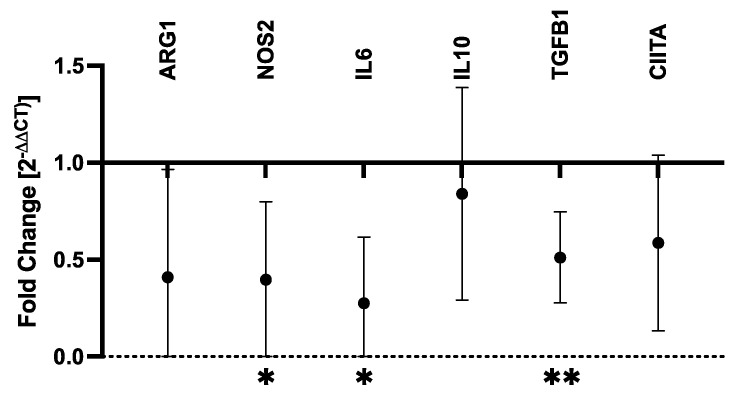
Fold-change in mRNA transcripts in ATRA-treated vs. control canine MDMs. Canine PBMCs were differentiated into MDMs in the presence of rhM-CSF (25 ng/mL), and ATRA (2 μM) or vehicle control media for 5 days. Target gene expression was normalized to C12orf43 and fold-change (2^−^^ΔΔCt^) in ATRA-treated MDMs was compared to control MDMs. Log_2_(fold-change) was compared between ATRA-treated and control MDMs, and comparisons yielding *p*-values < 0.05 are shown. The mean and 95% confidence interval (CI) are shown. * *p* < 0.05; ** *p* ≤ 0.01; *n* = 4 per target gene.

RT-qPCR was performed to determine the effects of ATRA on proinflammatory (IL6, NOS2) and immunosuppressive (ARG1, IL10, TGFB1) soluble mediators as well as MHCII expression (CIITA). Control and ATRA-treated MDMs from six dogs were evaluated (Supplementary Table S1). ATRA exposure to differentiating MDMs significantly decreased NOS2 (mean fold-change = 0.40, *p* = 0.032), IL6 (mean fold-change = 0.28, *p* = 0.013), and TGFB1 (mean fold-change = 0.51, *p* = 0.003) mRNA expression (Figure 1). While not statistically significant, ARG1 and CIITA both displayed consistent and repeatable decreases in expression in ATRA-treated MDMs (ARG1: mean fold-change = 0.41, *p* = 0.057; CIITA: mean fold-change = 0.59, *p* = 0.055). We did not detect consistent changes in IL10 expression (mean fold-change = 0.84, *p* = 0.298).

**Figure 2:**
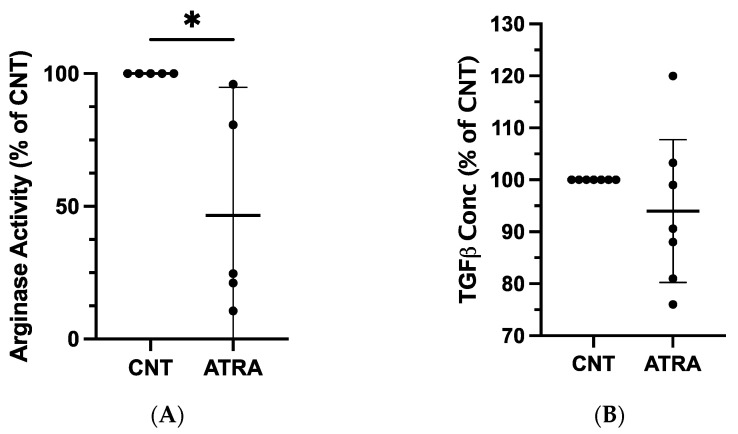
Evaluation of protein expression in ATRA-treated canine MDMs, as compared to control MDMs. Canine PBMCs were differentiated into MDMs in the presence of rhM-CSF (25 ng/mL), and ATRA (2 μM) or vehicle control media for 5 days. An equal number of ATRA-treated and control MDMs from each dog were input into each assay. (**A**) Arginase activity of control and ATRA-treated MDM lysates. *n* = 5. (**B**) TGF-β1 concentration in supernatant of control and ATRA-exposed MDMs after 24 h incubation. *n* = 7. Results are displayed as a percentage of paired control MDM values. The mean and 95% CI are shown, and individual data points are plotted. * *p* < 0.05.

We next determined whether the observed changes in mRNA expression could be detected at the protein level. An arginase activity assay performed on lysates from ATRA-exposed and control MDMs revealed repeatable and statistically significant decreases in arginase activity of ATRA-exposed MDMs (mean difference = −53%, *p* = 0.015; *n* = 5; Figure 2A). There were no consistent changes in TGF-β1 concentration in ATRA-treated vs. control MDMs (mean difference = −6%, *p* = 0.305; *n* = 7; Figure 2B).

### 3.3. Phenotypic Effects of ATRA on Differentiating Canine MDMs

**Figure 3:**
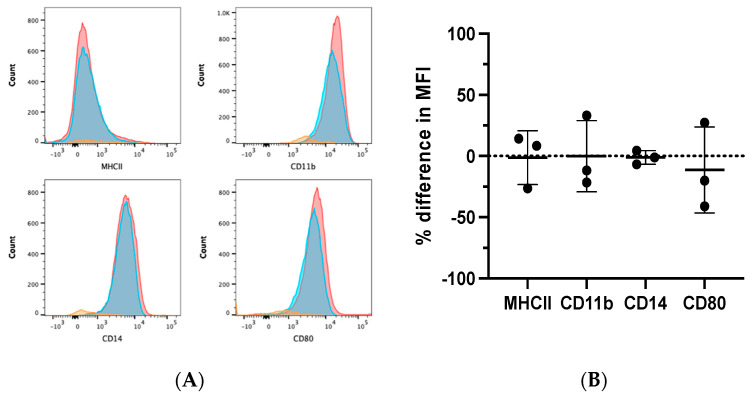
Phenotypic effects of ATRA on differentiating canine MDMs. Canine PBMCs were differentiated into MDMs in the presence of rhM-CSF (25 ng/mL), and ATRA (2 μM) or vehicle control media for 5 days. MDMs were analyzed after first gating on the myeloid cell cloud, single cells, and CD5− cells (Supplementary Figure S1A). (**A**) Representative histograms illustrating MHCII, CD11b, CD14, and CD80 expression in control (red) and ATRA-treated (blue) MDMs. Negative expression controls (orange) consisted of FMOs for CD11b and CD80, and CD5+ INCs for MHCII and CD14. (**B**) Summary of MCHII, CD11b, CD14, and CD80 MFI in ATRA-treated MDMs, relative to control MDMs. Mean and standard deviation are shown, and individual data points are plotted. *n* = 3.

Flow cytometry was performed on ATRA-treated and control MDMs from three dogs to evaluate the differential expression of phenotypic markers of monocytes/macrophages, such as CD11b, CD14, MHCII, and CD80 [7,11,29]. MDMs were determined to be positive for CD11b, CD80, and CD14 expression, and negative for MHCII expression. ATRA treatment during MDM differentiation did not alter this pattern of expression (Figure 3A,B). 

### 3.4. Effects of Arginase on GZMB Expression by Canine CD8+ T-Lymphocytes

**Figure 4:**
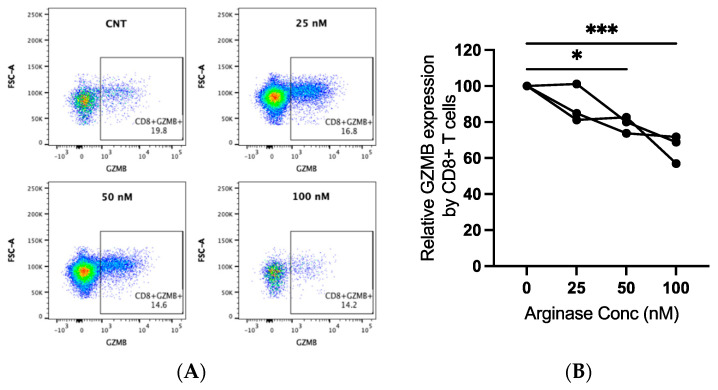
Granzyme B (GZMB) expression in canine CD8+ T-lymphocytes exposed to arginase. PBMCs from three dogs were incubated in complete media, with or without indicated concentrations of arginase (25–100 nM), for two days prior to flow cytometry analysis. CD8+ T-lymphocytes were analyzed after first gating on the lymphocyte cloud, single cells, live cells, CD3+, and CD4−CD8+ cells (Supplementary Figure S1B). Representative scatter plots (**A**) and summary graph (**B**) show the proportion of GZMB+ CD8+ T-lymphocytes with increasing concentrations of arginase. Lines connect data points from each individual dog. * *p* < 0.05; *** *p* < 0.001; *n* = 3.

Canine PBMCs from three individual dogs were incubated in arginase at the indicated concentrations for two days prior to the evaluation of GZMB expression by flow cytometry. We detected significantly decreased GZMB expression by CD8+ T-lymphocytes with increasing concentrations of arginase. The GZMB expression decreased by an average of 21% at 50 nM (*p* = 0.015) and 34% at 100 nM (*p* < 0.001; Figure 4A,B). Arginase did not induce consistent changes in CD3 expression on lymphocytes (data not shown).

### 3.5. Effects of Arginase on Proliferating Canine CD4+ & CD8+ T-Lymphocytes 

**Figure 5:**
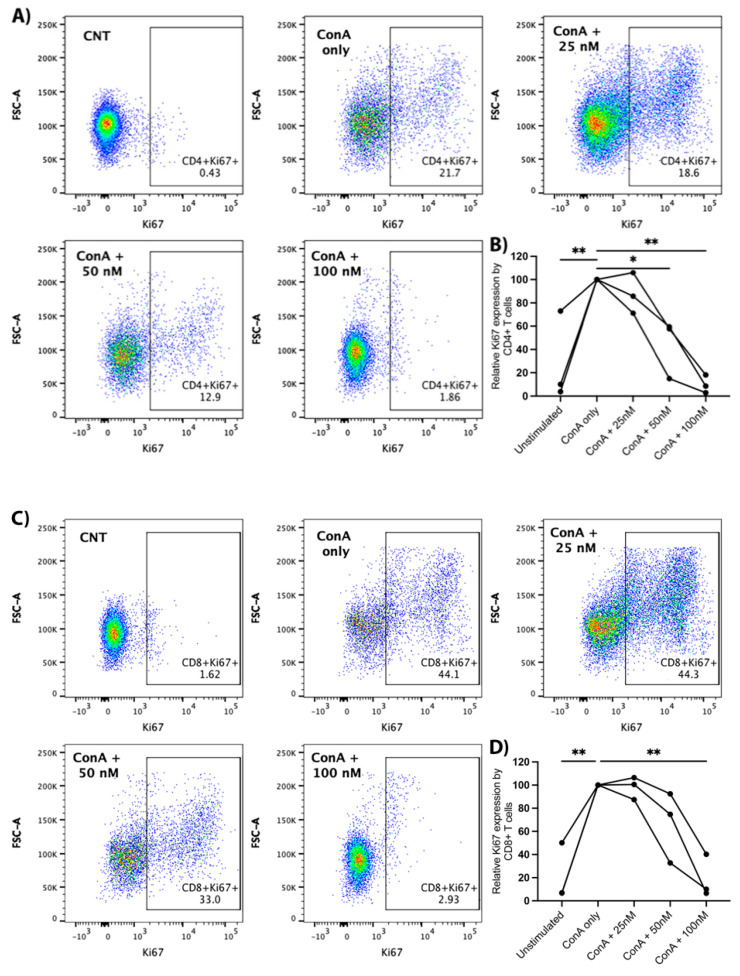
Proliferative effects of arginase on CD4+ and CD8+ T-lymphocytes. PBMCs from three dogs were incubated in complete media, complete media with ConA (5 μg/mL), or complete media with ConA (5 μg/mL) and indicated concentrations of arginase (25–100 nM) for two days prior to flow cytometry analysis. Cells were gated on the lymphocyte cloud, single cells, live cells, CD3+, and CD4+CD8− or CD4−CD8+ (Supplementary Figure S1C). Representative scatter plots (**A**), and summary graph (**B**) show the proportion of Ki67+ CD4+ T-lymphocytes treated with/without ConA and increasing concentrations of arginase. Representative scatter plots (**C**) and summary graph (**D**) show the proportion of Ki67+ CD8+ T-lymphocytes treated with/without ConA and increasing concentrations of arginase. Lines connect data points from each individual dog. * *p* < 0.05; ** *p* ≤ 0.01; *n* = 3.

Canine PBMCs were treated with ConA (5 μg/mL) and arginase at the indicated concentrations for two days prior to the evaluation of Ki67 expression with flow cytometry. We observed a significant decrease in Ki67 expression with increasing concentrations of arginase for CD4+ and CD8+ T-lymphocytes. Proliferation was significantly decreased at arginase concentrations of 50 nM (mean difference = −56%, *p* = 0.036) and 100 nM (mean difference = −90%, *p* = 0.002) in CD4+ T-cells (Figure 5A,B), and at an arginase concentration of 100 nM (mean difference = −81%, *p* = 0.002) in CD8+ T-cells (Figure 5C,D). Arginase did not induce consistent changes in CD25 expression on mitogen-stimulated lymphocytes (data not shown).

## 4. Discussion

The TME comprises several immunosuppressive myeloid cells and soluble mediators that contribute to tumor progression. The modification of the TME may enhance anti-tumor immunity, making it an attractive therapeutic strategy. We therefore aimed to explore the ability of ATRA to alter canine MDMs in vitro. We observed consistent 2–4-fold decreases in arginase, iNOS, IL-6, TGF-β1, and CIITA mRNA transcripts in MDMs exposed to non-cytotoxic doses of ATRA. Arginase activity was also consistently decreased in ATRA-treated MDMs, as compared to vehicle controls. Finally, we showed that arginase exerted significant negative effects on T-lymphocyte proliferation and the expression of GZMB, a critical mediator of cellular cytotoxicity. Altogether, these findings suggest the potential of ATRA to reverse the inhibitory effects of MDM-derived arginase on canine T-lymphocyte function. However, further studies are necessary to establish a direct link between ATRA’s effects on canine MDSCs and T-cell function.

Our findings of decreased pro-inflammatory (IL-6, iNOS) and immunosuppressive (arginase, TGF-β1) mRNA transcripts and reduced arginase activity in ATRA-treated MDMs, suggested that ATRA may be capable of dampening the immunomodulatory effects of canine macrophages. The lack of a statistically significant difference in ARG1 expression between the control and ATRA-treated MDMs (*p* = 0.057), despite statistically significantly decreased arginase activity (*p* = 0.015) in ATRA-treated MDMs, could be indicative of a type II error or, alternatively, evidence of a post-translational inhibition of arginase by ATRA. These findings were mostly consistent with a previous report in which the ATRA treatment of human monocyte-derived MDSCs in vitro significantly reduced mRNA expression of TGF-β [21]. We did not, however, identify a significant decrease in IL-10, as reported by Tobin et al. [21]. Similar findings were also reported by Shao et al., who showed that ATRA inhibited M2 phenotypic features (TGF-β and CD209 expression) in RAW264.7 macrophages [20]. Interestingly, two prior publications reported that ATRA increased arginase expression by murine bone-marrow-derived macrophages and dendritic cells [22,23]. While this was seemingly in conflict with our results, the discrepancy was attributed to the different conditions for in vitro differentiation, with both of these studies utilizing mouse bone marrow cells cultured with IL-4 and including either GM-CSF or L-929 fibroblast-conditioned media (as a source of M-CSF). Additionally, both studies used lower doses of ATRA (50–100 nM). Our findings suggested that ATRA may be capable of suppressing the immunomodulatory capabilities of canine macrophages. However, previous reports have suggested that ATRA may have differing effects on other cell subsets, which thus necessitates the evaluation in vivo in order to determine the suitability of ATRA as an immunotherapeutic for canine cancer.

In contrast to previous reports in humans and mice [18,19,20,21], we found that ATRA was unable to alter the phenotype of canine differentiating macrophages. In our study, ATRA-treated canine MDMs displayed consistent, albeit statistically insignificant, decreases in transcripts of a positive regulator of MHCII (CIITA). In several reports, ATRA increased the expression of MHCII and mature myeloid cell markers, revealing its differentiating abilities [18,19,20,21]. A lack of MHCII and costimulatory molecule expression was a common finding of immunosuppressive myeloid cells in tumor-bearing patients. The absence of these molecules impairs these cells in their ability to stimulate T-lymphocytes, further promoting tumor progression [16]. In our study, canine MDMs were negative for MHCII in both the control and ATRA-treated samples. Canine MDMs have been either positive [30], or nearly negative [31], for MHCII in previous reports, with expression likely depending on differentiation conditions that varied between studies. Our differentiation conditions were similar to those reported by Hartley et al. [25], although their MDM phenotype was not reported. Although the ATRA treatment of canine MDMs did not upregulate MHCII expression in our study, it also did not alter the expression of CD11b, CD14, or the costimulatory molecule, CD80. Similar findings have been reported in other cell types exposed to ATRA, whereby cell function was altered in spite of the stable expression of CD11b, CD14, and costimulatory molecules [21,22]. Therefore, ATRA treatment may diminish the MDM expression of immunosuppressive soluble mediators while maintaining the cell surface expression of receptors such as CD80 that facilitate the adaptive immune response. 

We have also shown that increasing arginase concentration was correlated with the decreasing proliferation of both CD4+ and CD8+ canine T-lymphocytes and decreasing GZMB expression by CD8+ T-lymphocytes. GZMB is a protease found in the granules of cytotoxic T-lymphocytes that induces apoptosis in target cells, and it has commonly served as a marker of cytotoxic T-lymphocytes [32]. These findings were consistent with previous studies in humans and mice that revealed decreased or restored T-lymphocyte function through the depletion or addition of L-arg, respectively [1,2,3,4,5]. The production of arginase by immunosuppressive myeloid cells is a major mechanism of T-lymphocyte impairment within the TME and can facilitate enhanced tumor progression [3,4,6]. Numerous studies in humans and mice have demonstrated increased concentrations of arginase in patients with cancer, as compared to healthy controls, and the inhibitory effects of arginase on T-lymphocyte function [3,4,5,6,33]. For example, the activated macrophages producing arginase in mice were shown to inhibit T-lymphocyte function by decreasing the proliferation and expression of the CD3ζ chain of the T-cell receptor (TCR) [5]. Furthermore, MDSCs from humans with gastric cancer were shown to suppress CD8+ T-lymphocyte function by decreasing the IFN-γ and GZMB expression via arginase production [33]. Arginase has previously been evaluated in only one canine study where it was shown to be expressed by MDSCs in tumor-bearing dogs via RT-qPCR, but the direct effect of arginase on canine lymphocytes was not evaluated [7]. Our findings confirmed similar inhibitory effects of arginase on canine T-lymphocytes, as compared to other species. 

The study reported herein detailed the effects of ATRA on canine MDMs; ATRA decreased arginase activity in MDMs, and arginase inhibited canine T-lymphocyte functions. Moreover, ATRA may have the potential to enhance T-lymphocyte function in microenvironments containing high arginase activity due to macrophage arginase expression. However, additional previously reported effects of ATRA included the stabilization of regulatory T-cell functions within an inflammatory milieu, the activation of dendritic cells and the generation of effector T-cells, the differentiation of B cells into antibody-producing plasma cells, the induction of lymphocyte homing to the gut, and the inhibition of macrophage expression in inflammatory mediators [34,35,36,37]. Therefore, it is critical to interpret our findings with caution. In vivo studies will be necessary to determine the net effect of ATRA on canine T-lymphocyte function within a complex tumor microenvironment. 

Limitations of this study included the relatively small number of biological replicates and the limited number of target genes and proteins evaluated, as well as that the ATRA effects on MDMs were only evaluated for one concentration. Unfortunately, due to the variable small amount of MDMs retrieved per blood sample, the authors had to be selective with the assays performed and the number of biological replicates evaluated. While we may have been able to retrieve more blood and larger amounts of MDMs from younger, healthy laboratory dogs, our sample collection ensured our results were representative of a population of cancer-bearing dogs and were, therefore, clinically translatable. 

## 5. Conclusions

We demonstrated that arginase dampened canine T-lymphocyte function in addition to showing preliminary evidence that ATRA may be able to serve as a tool to modify immunosuppressive and proinflammatory capabilities of canine macrophages. The potential for ATRA as a canine cancer treatment cannot be determined without further in vivo clinical studies to evaluate the effects of ATRA on immunosuppressive myeloid cells within the complex TME of an immunocompetent canine host. 

## Data Availability

Data supporting reported results can be found herein, in Supplementary Materials, and upon request to the corresponding author (S.S.W.).

## Supplementary Materials

The following supporting information can be downloaded at: https://www.mdpi.com/article/10.3390/vetsci9070374/s1, Figure S1: Flow cytometry gating strategies; Figure S2: Effect of ATRA on the viability of canine MDMs; Table S1: Clinical characteristics of dogs included; Table S2: Antibodies utilized; Table S3: Primers utilized for RT-qPCR studies; Table S4: Descriptive statistics and *p*-values for all comparisons.
