# Supplementary material for: The Effect of Arginase on Canine T-Lymphocyte Functions and its Modulation by All-Trans Retinoid Acid (ATRA) in Canine Monocyte-Derived Macrophages
Source: Vet Sci. 2022 Jul 21;9(7):374. doi: 10.3390/vetsci9070374 (PMC9320773; doi:10.3390/vetsci9070374)
Supplement: Supplementary file 1 [file vetsci-09-00374-s001.zip › vetsci-1809858-Supplementary Figures.pdf]

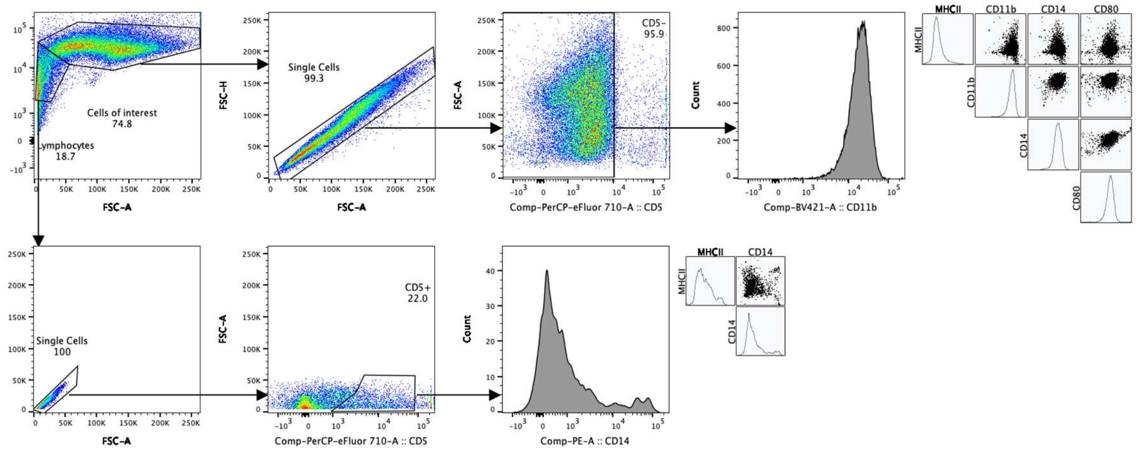

SSC-A
FSC-A
Cells of interest
74.8
Lymphocytes
18.7
FSC-H
Single Cells
99.3
FSC-A
CD5-
95.9
Comp-PerCP-eFluor 710-A :: CD5
Count
Comp-BV421-A :: CD11b
MHCII
CD11b
CD14
CD80
Single Cells
100
CD5+
22.0
Comp-PerCP-eFluor 710-A :: CD5
Comp-PE-A :: CD14

(A)

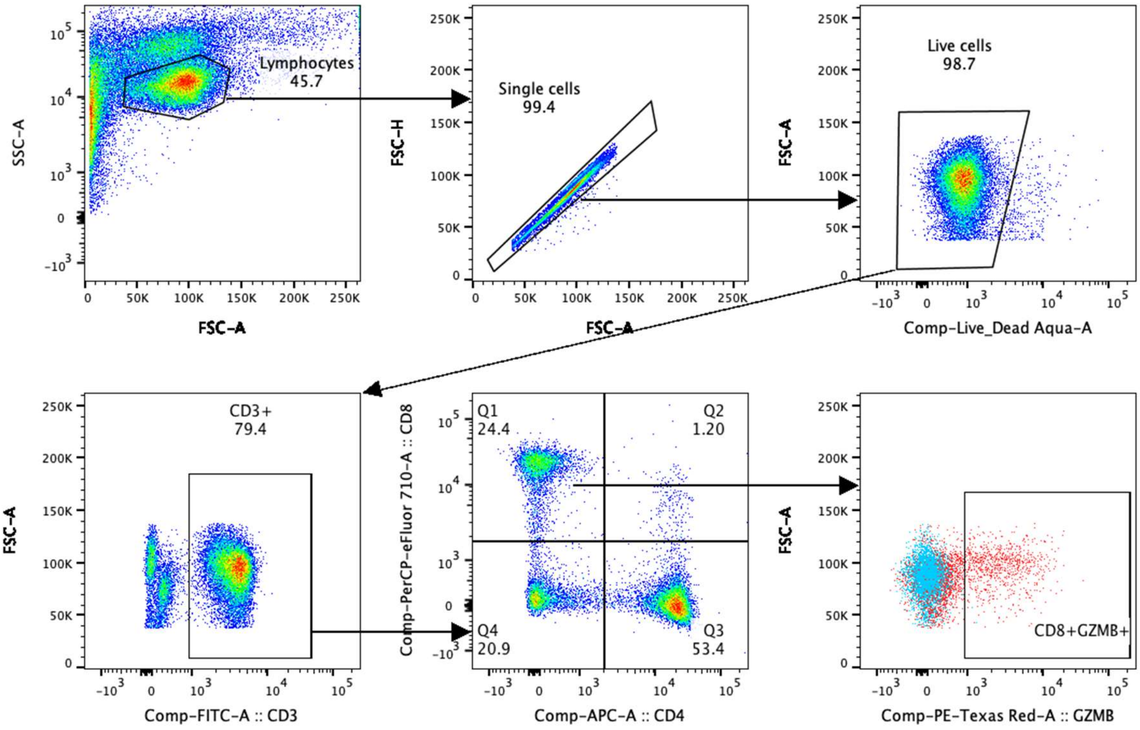

SSC-A
Lymphocytes
45.7
FSC-A
FSC-H
Single cells
99.4
Live cells
98.7
Comp-Live_Dead Aqua-A
CD3+
79.4
Comp-FITC-A :: CD3
Comp-PerCP-eFluor 710-A :: CD8
Q1
24.4
Q2
1.20
Q3
53.4
Q4
20.9
Comp-APC-A :: CD4
CD8+GZMB+
Comp-PE-Texas Red-A :: GZMB

(B)

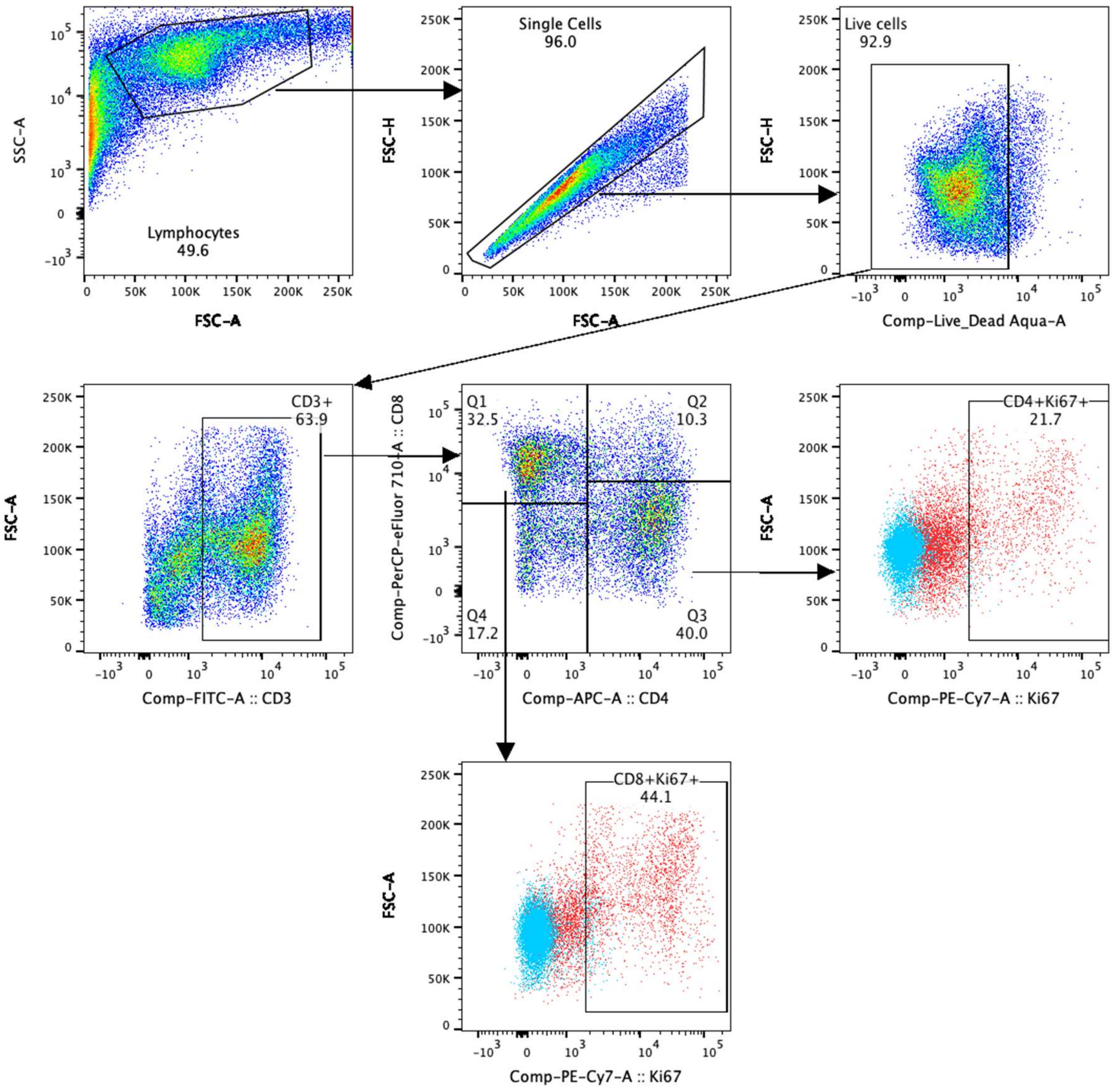

(**C**)

Supplemental Figure S1. Flow cytometry gating strategies. A) Gating strategy used for evaluation of canine MDM phenotype. Cells were gated on the myeloid cell cloud, single cells, and CD5⁻ cells. CD5+ cells were used as INCs to establish gating for MHCII and CD14. B) Gating strategy used for evaluation of GZMB expression by CD8+ T-lymphocytes exposed to arginase. Cells were gated on the lymphocyte cloud, single cells, live cells, CD3+, and CD4-CD8+ cells. In the final panel, the FMO control (blue) is overlaid onto a fully stained sample (red). C) Gating strategy used for evaluation of Ki67 expression by CD4+ and CD8+ T-lymphocytes exposed to ConA and arginase. Cells were gated on the lymphocyte cloud, single cells, live cells, CD3+, and CD4+CD8- or CD4-CD8+ cells. In the final two panels, the unstimulated control (blue) is overlaid onto a ConA-stimulated sample (red).

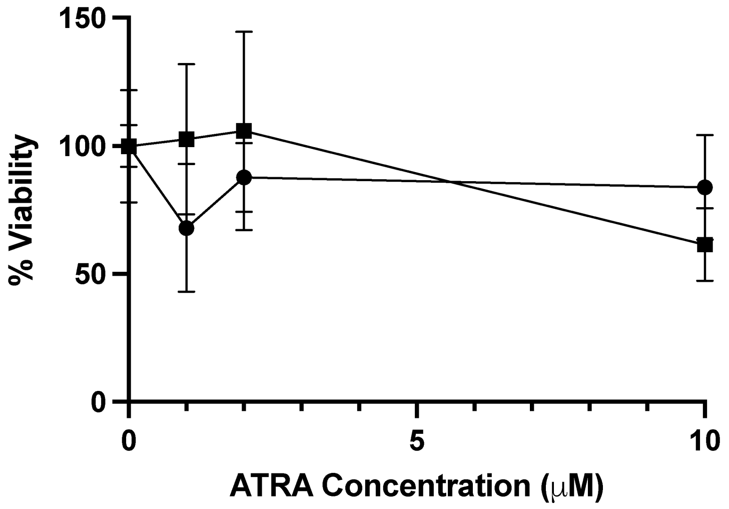

Supplemental Figure S2. Effects of ATRA on the viability of canine MDMs. MDMs from two dogs were differentiated in rhM-CSF (25 ng/ml) for five days with increasing concentrations of ATRA (1 – 10 μM) or DMSO only. Results are expressed as % viability relative to DMSO control. Lines connect data points from each individual dog. Mean and standard deviation are shown.
