# Supplementary material for: The Effect of Arginase on Canine T-Lymphocyte Functions and its Modulation by All-Trans Retinoid Acid (ATRA) in Canine Monocyte-Derived Macrophages
Source: Vet Sci. 2022 Jul 21;9(7):374. doi: 10.3390/vetsci9070374 (PMC9320773; doi:10.3390/vetsci9070374)
Supplement: Supplementary file 1 [file vetsci-09-00374-s001.zip › vetsci-1809858-Supplementary Table S1.pdf]

Supplementary Table S1. Clinical characteristics of dogs included.

Age (yrs)	Sex/neuter status	Breed	Weight (kg)	Disease
			MDM RTqPCR	
9	MN	GSD	46	Multicentric B cell lymphoma
9	FS	Labrador retriever	38	Multicentric B cell lymphoma
10	MC	Labrador retriever	34	Vertebral mass of unknown etiology
4	MC	Mixed	32	Multicentric lymphoma of unknown phenotype
13	MN	Mixed	32	Lipomas
11	FS	Mixed	27	Multicentric B cell lymphoma
			MDM arginase assay	
9	FS	Labrador retriever	32	Oral malignant melanoma
9	MC	Weimaraner	48	Meningioma
10	MC	Mixed	35	Metastatic prostate carcinoma*
12	MC	Welsh terrier	13	Metastatic pulmonary carcinoma*
11	MC	Pitbull	29	Multicentric B cell lymphoma
11	MC	Golden retriever	46	Liposarcoma
			MDM ELISA	
12	MC	Border Collie	32	Multicentric B cell lymphoma
11	MC	Pitbull terrier	30	Metastatic osteosarcoma
9	MC	Mixed	29	Metastatic prostate carcinoma
14	FS	Mixed	23	Oral carcinoma (suspect salivary origin)
8	MC	Labrador retriever	24	Multicentric B cell lymphoma
13	F	Maltese terrier	3	Multicentric B cell lymphoma and multiple mammary tumors
9	MC	Mixed	31	Multicentric B cell lymphoma
			MDM flow cytometry	
12	FS	Pit Bull terrier	23	Multicentric B cell lymphoma
6	FS	Boxer	28	Mast cell tumor
11	FS	Mixed	27	Multicentric B cell lymphoma
		Changes in phenotype and proliferation of canine PBMCs exposed to arginase		
2	FS	Labrador retriever	30	Metastatic mast cell tumor
10	MC	Labrador retriever	27	Multiple cutaneous mast cell tumors
8	MC	GSD	33	Multicentric B cell lymphoma

* Control and ATRA-treated MDMs from these dogs were combined to provide sufficient numbers to input into the arginase activity assay

F = intact female; FS = female spayed; MC = male castrated

GSD = German shepherd dog
