# Supplementary material for: The Effect of Arginase on Canine T-Lymphocyte Functions and its Modulation by All-Trans Retinoid Acid (ATRA) in Canine Monocyte-Derived Macrophages
Source: Vet Sci. 2022 Jul 21;9(7):374. doi: 10.3390/vetsci9070374 (PMC9320773; doi:10.3390/vetsci9070374)
Supplement: Supplementary file 1 [file vetsci-09-00374-s001.zip › vetsci-1809858-Supplementary Table S2.pdf]

Supplementary Table S2. Antibodies utilized.

Antigen/marker	Conjugated fluorochrome	Antigen species	Antibody clone #	Manufacturer	Catalogue #	Dilution
			Cell Surface			
Viability dye	Aqua	N/A	N/A	Thermo Fisher	L34966	1:1000
Fc block	N/A	Canine	N/A	eBioscience	14-9162-41	1:5
CD4	APC	Canine	YKIX302.9	Invitrogen	17-5040-42	1:10
CD8	PerCp-Cy5.5	Canine	YCATE55.9	eBioscience	46-5080-41	1:25
CD25	PE	Canine	P4A10	eBioscience	12-0260-042	1:10
CD5	PerCp-ef710	Canine	YKIX322.3	eBioscience	5016058	1:10
CD11b	Biotin	Canine	CA24.3E4	Moore lab	BDB563259	1:50
Streptavidin	BV421	N/A	N/A	BD Biosciences	563259	1:50
CD14	PE	Human	TUK4	Invitrogen	MHCD1404	1:25
MHCII	APC	Canine	YKIX334.2	Thermo Fisher	17-5909-42	1:10
CD80	PE-CF594	Murine	16-10A1	Thermo Fisher (BD)	BDB562504	1:25
			Intracellular			
CD3	FITC	Human	CD3-12	eBioscience	MCA1477F	1:50
Granzyme B	PE-TR	Human	GB11	Invitrogen	GRB17	1:50

Ki67	PE-Cy7	Canine/Human	20Raj1	eBioscience	25-5699-42	1:10
