# Supplementary material for: The Effect of Arginase on Canine T-Lymphocyte Functions and its Modulation by All-Trans Retinoid Acid (ATRA) in Canine Monocyte-Derived Macrophages
Source: Vet Sci. 2022 Jul 21;9(7):374. doi: 10.3390/vetsci9070374 (PMC9320773; doi:10.3390/vetsci9070374)
Supplement: Supplementary file 1 [file vetsci-09-00374-s001.zip › vetsci-1809858-Supplementary Table S3.pdf]

Supplementary Table S3. Primers utilized for RT-qPCR studies.

Gene	Forward primer (5'- 3')	Reverse primer (3'- 5')	RefSeq#	Product size (bp)
IL10	CAAGCCCTGTCGGAGATGAT	TGATGTCTGGGTCGTGGTTC	NM_001003077.1	76
ARG1	ACCCATCTCTGGGGAAGACA	CCGAAGCAAGCCAGGGTTAT	XM_532053.7	79
NOS2	CACGTTTGGGAACGGAGACT	CTATTGGGGCGAGCTGAGAG	NM_001313848.1	197
TGFB1	ACCACGCTAACTTCTGCCTG	ACGTAGTACACGATGGGCAG	NM_001003309.1	160
IL6	AGTGACCACTCCTGACCCAA	GTGCTTCACGCACTCATCCT	NM_001003301.1	76
CIITA	CTGGAGCTGCTGCACTGT	GTCCAGGGAGAAGTCTTGGC	XM_038669177.1	168
C12orf43	AATTCCGAGCCCACGTAGC	CTCAGCCTTTCTCAGCTCCT	XM_849238.1	92
RPS5	GAAGACTCAACCCGCATTGG	CCTGATTCACACGGCGTAG	XM_038655656.1	82
